# Emotional, Ethical and Cultural Challenges in Percutaneous Endoscopic Gastrostomy (PEG) Decision‐Making: A Systematic Review and Meta‐Synthesis

**DOI:** 10.1111/hex.70294

**Published:** 2026-04-24

**Authors:** Hande Nur Arslan, Gamze Bozkul, Sevilay Şenol Çelik

**Affiliations:** ^1^ Department of Surgical Nursing School of Nursing, Koc University Istanbul Türkiye; ^2^ Nursing Department, Faculty of Health Sciences Tarsus University Mersin Türkiye

**Keywords:** meta‐synthesis, percutaneous endoscopic gastrostomy, systematic review

## Abstract

**Background:**

Percutaneous endoscopic gastrostomy (PEG) is a critical intervention for patients with neurological and gastrointestinal conditions affecting oral intake. While clinical guidelines emphasise medical indications, they often overlook the intricate emotional, ethical and cultural concerns that shape decision‐making. This gap in understanding leads to variability in clinical recommendations and uncertainty among patients and their families. A deeper exploration of these factors is necessary to support informed, patient‐centred decision‐making.

**Aim:**

This systematic review and meta‐synthesis aimed to explore the emotional, ethical and cultural challenges influencing PEG decision‐making, while also considering the broader context of shared decision‐making.

**Design:**

A systematic review and meta‐synthesis of qualitative studies.

**Methods:**

The review was conducted by systematically searching six databases, including CINAHL, Scopus, Web of Science, MEDLINE, PubMed and TRDizin. Only qualitative studies published between 2004 and 2024 were included to capture subjective experiences related to PEG decision‐making. Studies focusing only on clinical outcomes or utilising quantitative methodologies were excluded. The review considered perspectives from adult patients, family members, caregivers and healthcare professionals while paediatric studies were excluded due to differences in decision‐making dynamics. Data were synthesised using thematic analysis to organise findings into main themes and sub‐themes.

**Results:**

A total of 15 studies representing a variety of clinical settings and patient conditions, such as amyotrophic lateral sclerosis, advanced dementia and stroke, were included. These studies involved 141 patients (41.1%), 62 caregivers (18.1%) and 140 healthcare professionals (40.8%), ensuring a comprehensive analysis of perspectives on PEG decision‐making. Seven major themes were identified: (1) emotional and psychological impact of decision‐making, (2) ethical and moral considerations both patients and caregivers, (3) communication challenges and information gaps, (4) impact of healthcare professionals on decision‐making, (5) ethical and emotional challenges in decision‐making, (6) communication barriers and conflicting advice and (7) professional responsibility and advocacy. Family members and caregivers reported feelings of anxiety, guilt and regret, often due to uncertainty and inadequate communication. Healthcare professionals also faced challenges, including conflicting messages and a lack of comprehensive information.

**Conclusion:**

Emotional, ethical and cultural factors significantly impact the PEG decision‐making process involving patients, caregivers and healthcare professionals. Improving healthcare professionals' communication skills, developing decision aids and encouraging interdisciplinary collaboration are crucial for supporting informed and shared decision‐making.

**Implications for Nursing:**

Nurses play a central role in the PEG decision‐making process, as they are the healthcare professionals with the most frequent and direct contact with patients and their family support networks. Addressing the gaps in communication and emotional support can help improve the quality of care provided to patients undergoing PEG. Implementing structured emotional support programmes, integrating psychological counselling into routine care and training healthcare professionals in empathetic communication strategies can significantly reduce patient and caregiver distress. Beyond providing clinical care, nurses act as essential advocates, educators and emotional support providers, ensuring that patients and families receive clear, consistent and compassionate guidance throughout the decision‐making process. Their involvement in interdisciplinary collaboration and shared decision‐making frameworks is crucial for aligning PEG decisions with patient values and preferences.

**Patient and Public Contributions:**

This review synthesised findings from studies capturing the experiences of patients, families, caregivers and healthcare professionals involved in PEG decision‐making, ensuring their perspectives were represented.

## 1 Introduction

Percutaneous endoscopic gastrostomy (PEG) has become a critical intervention in the clinical management of patients with conditions that significantly impair oral intake. This procedure is indicated in many critically ill patients, particularly those with neurodegenerative disorders such as amyotrophic lateral sclerosis (ALS), advanced dementia, stroke‐related dysphagia and head and neck malignancies [1, 2]. These conditions often lead to severe nutritional deficiencies, and the use of a PEG tube provides a viable solution that bypasses the oropharyngeal route, reducing the risk of malnutrition, dehydration and associated morbidity [3, 4].

Although it is a procedure with significant clinical benefits, the choice to place a PEG involves ethical, emotional, cultural and logistical dimensions that go beyond medical issues. The decision‐making process can be burdened by patient and family anxiety, uncertainty about prognosis, communication gaps between healthcare professionals and families, and ethical dilemmas regarding quality of life and end‐of‐life care. Given its end‐of‐life implications, PEG decisions should ideally factor in additional variables—including a long‐term prognosis of the patient's overall health, quality of life and benefits balanced against burdens [5]. Unlike other medical care forms, PEG often marks a critical transition in a patient's trajectory, indicating a shift from short‐term nutritional support to long‐term enteral feeding. For many patients, this decision is associated with disease progression, loss of oral feeding ability and increased dependency on caregivers and medical support. It can be considered as marking the shift from curative to palliative management, as families and caregivers often perceive PEG as a means of life prolongation rather than a therapeutic intervention. Studies have shown that in conditions like ALS and advanced dementia, PEG is frequently associated with comfort care rather than recovery, contributing to complex emotional and ethical dilemmas during decision‐making [6, 7]. This change can dramatically alter the patient's disease perception and that of their family, initiating difficult questions regarding the goal of care and for whom [8]. In addition, PEG is associated with potential complications/risks for the individual/caregivers and associated care burden. Boylan et al. [9] reported that 16.7% experienced a complication within the first 30 days after PEG, and the most commonly reported complication was peristomal pain (9.2%). After 1 year, the most common complications were pain (14.4%), followed by weeping at the site, site infection and external hypergranulation [9]. Özceylan ve Findik [10] reported that although the duration of hospitalisation was longer, a decrease in anxiety and depression was observed in the caregivers of patients receiving PEG compared to the other feeding methods group, and the decrease in anxiety and depression levels was less pronounced as the age of the caregiver increased [10]. Thus, the recommendation for PEG should be made by a multidisciplinary team (doctors, nurses, physiotherapists, speech therapists and dietitians) based on clinical, ethical, nutritional and swallowing function assessments. The final decision should be made collaboratively by the patient, their family and the care team, ensuring shared decision‐making that aligns with patient values and preferences.

Most of the time, primary decision‐makers are relatives who are also caregivers; this situation can bring severe moral distress, especially if there are no clear indications about what the patient would have wanted or when family members disagree [11]. Moreover, cultural factors make the decision‐making process even more difficult. Decisions regarding PEG can be significantly affected by the culture's beliefs on autonomy, reverence for life and the validity of medical actions [12]. In other words, some societies value using all medical techniques to maintain the life of an individual, while others may approve of natural death without technological support. Consequently, different cultures hold different perspectives that might lead to conflicts between healthcare providers and families, requiring skilful management before reaching an agreement that respects patient values and clinical facts [13]. Gieniusz et al. [14] emphasised that decisions about enteral nutrition should be provided individually, with the patient's wishes thoroughly discussed and incorporated into the decision‐making process. The American Society for Parenteral and Enteral Nutrition (ASPEN) supports this sentiment, emphasising that nurses play a key role in providing patients with evidence‐based literature to help them make informed decisions [15]. To actively participate in this process, patients must have access to relevant evidence during the PEG decision‐making process to ensure informed choices that align with their values and medical needs [16]. However, the available literature and expert opinions are not always consistent, leading to uncertainties in treatment planning and decision‐making.

Evidence suggests that the process can be afflicted by communication challenges and insufficient dissemination of information, leading to a misalignment between patient and family expectations and the reality of PEG outcomes. While many expect PEG to improve quality of life or restore normal feeding, they often face unexpected complications, increased dependency and prolonged end‐of‐life care, contributing to emotional distress and ethical dilemmas [17]. A central issue in decision‐making is the communication process between healthcare professionals and caregivers. Healthcare providers must communicate effectively with the patient, using an understanding of their medical condition to correctly explain all risks and benefits associated with PEG. However, scientific studies share consistent findings indicating that communication is often imperfect and associated with misinformation, decisional conflict or regret [18, 19]. For example, repeated studies have demonstrated that caregivers often report feeling inadequately informed regarding potential complications such as infection and long‐term care [14, 20]. In addition, there is limited reported management discussion around nutritional interventions that enable continued use of a nasogastric tube or palliative feeding approaches, which may more closely reflect the patient's overarching goals of care [16]. In addition to the gaps in information, the emotional weight of deciding on a PEG tube cannot be underestimated. It is a stressful process for patients and caregivers as they determine if it is right to keep them alive or not while considering the possible lowering of their quality of life [21].

For patients with progressive and terminal medical conditions like advanced dementia or ALS, PEG may involve broader end‐of‐life issues [22]. At this stage, an ethical dilemma arises regarding life‐sustaining measures: Should the focus remain on extending the patient's life or shift towards enhancing comfort and preserving dignity during the remaining time? [15]. The complexity of these decisions illustrates the importance of adopting a patient‐centred approach, which combines medical evidence with patients' values, preferences and goals. To enhance decision‐making processes, it is crucial to have an understanding of what people go through during PEG. Considering this background, the systematic review summarises qualitative research on decision‐making regarding PEG. The information will come from analysing real‐life experiences of patients, family members taking care of them and healthcare team who deal with them. In the literature, no systematic review and meta‐synthesis study examining the emotional, ethical and cultural challenges in the PEG decision‐making process could be found. Therefore, this study identifies common difficulties that patients face when making decisions, their need for information and the ethical issues that arise from this procedure. This systematic review and meta‐synthesis aimed to explore the emotional, ethical and cultural challenges influencing PEG decision‐making, while also considering the broader context of shared decision‐making. Additionally, this study seeks to support reflective thinking among healthcare providers, patients and their families to promote informed and compassionate decision‐making during PEG placement.

### 1.1 Study Questions

1. What are the emotional challenges in PEG decision‐making?
2. What are the ethical challenges in PEG decision‐making?
3. What are the cultural challenges in PEG decision‐making?

## 2 Methods

### 2.1 Study Design

The study was conducted using a systematic review and meta‐synthesis design for qualitative studies. The present study followed the Preferred Reporting Items for Systematic review and Meta‐Analysis Protocols (PRISMA) 2020 [23].

### 2.2 Selection Criteria and Literature Search

Several databases were searched: CINAHL, Scopus, Web of Science, MEDLINE, PubMed and TRDizin. The search terms were a combination of Medical Subject Heading (MeSH) terms and text words like ‘decisional conflict’, ‘decision‐making’, ‘enteral nutrition’, ‘feeding tube’ and ‘percutaneous endoscopic gastrostomy’. The literature revealed standard search terms and phrases used in clinical practice. The time from 2004 to 2024 was chosen to encompass the most recent and relevant literature. The article screening process was conducted using Covidence, a web‐based platform designed to streamline systematic reviews (Covidence, Veritas Health Innovation, Melbourne, Australia. www.covidence.org [24]). Covidence was used to automatically detect and remove duplicate records, facilitate blinded title and abstract screening by two independent reviewers, and manage full‐text eligibility assessments. Conflicts between reviewers were resolved through discussion, ensuring a rigorous and unbiased selection process.

### 2.3 Inclusion and Exclusion Criteria

Qualitative studies that focus on adults were included if they explicitly reported qualitative methodologies such as phenomenology, thematic analysis, grounded theory or narrative analysis. Mixed‐method studies were only included if qualitative data were separately reported and analysed. Only peer‐reviewed original research articles were included. Editorials, opinion pieces and non‐peer‐reviewed sources were excluded. If essential study details (e.g., methodology, participant characteristics, data collection or analysis methods) were missing, authors were contacted where possible. If no response was received or the information remained unclear, the study was excluded. Importantly, all studies included in this review, as well as the broader literature on this topic, specifically examined PEG decision‐making rather than alternative feeding tube methods. Caregivers were defined as individuals providing direct care and support to the patient, including spouses, children or professional home care providers. Family members referred to biological or legal relatives of the patient, while surrogates were those legally or ethically designated to make medical decisions on behalf of the patient. If the authors did not offer any explanations, the study was classified according to the kind of question it asked. If a study used open‐ended questions in focus groups or individual interviews to examine satisfaction or expectations, it was classified as qualitative. Research that only included children was excluded. Articles not in English or Turkish were excluded to prevent linguistic and cultural bias during translation.

### 2.4 Study Selection and Data Extraction

In this study, study selection was done in two steps. In the first stage, three independent researchers removed duplicate publications and evaluated their studies' titles and abstracts using the inclusion criteria. In the second stage, researchers independently assessed the entire text of the articles. When the researchers disagreed about the studies, the senior researcher (S.Ş.Ç.) was consulted, and a consensus was established. The researchers extracted data from the studies used in this systematic review, including study characteristics (author, year, country, study design, study sample, condition/setting and data collection) and results (primary themes, sub‐themes or main outcomes) (Table 1).

**Table 1:** Summary of key features and main conclusions of the articles included in the systematic review and meta‐synthesis.

Author–year–country	Study design	Study sample	Condition	Data collection	Themes and sub‐themes (for qualitative studies)	Main outcomes
Brotherton and Abbott [6], the United Kingdom	Qualitative study	16 Patients and 27 carers	Patients undergoing PEG	Semi‐structured interview	Themes/sub‐themes: *Patients* Poor communication Lack of information Inappropriate information Attitudes of healthcare professionals Exclusion *Carers* Lack of information Attitudes of healthcare professionals Body weight Before feeding: weight loss Post feeding: weight gain There was simply ‘no alternative’	The results showed that the decision‐making process about PEG feeding was complicated most by the need for more effective communication between healthcare professionals and patients. Ethical quandaries, such as reconciling the preservation of life considering the quality of life, add further complexity to these choices.
Clarke et al. [7], the United Kingdom	Qualitative study	29 Patients and their relatives	Patients undergoing PEG (amyotrophic lateral sclerosis, multiple sclerosis, Huntington's and dementia)	In‐depth qualitative interviews	Theme 1**:** Health literacy Theme 2**:** Planning style	The study revealed that patients and their families faced significant challenges dealing with the uncertainty associated with progressive neurological diseases. Health literacy considerably affected decision‐making, as more informed individuals reported feeling more in control.
Greenaway et al. [25], the United Kingdom	Qualitative study	21 Patients	Patients undergoing PEG (amyotrophic lateral sclerosis: ALS)	Semi‐structured interviews	Themes/sub‐themes: *Patient‐centric factors* Perceptions of choice and control Acceptance and need Aspects of fear *External factors* Health professionals: Doctors, nurses, therapists, care workers Family Information *The concept of time* Living in the moment ‘Right thing, right time’	The emotional burden of ALS, combined with the pressure of making life‐altering decisions such as gastrostomy, weighed heavily on patients and families. The study found that external influences, including the opinions of healthcare professionals, often compromised decision‐making autonomy.
[11], Australia	Qualitative study	32 Health professionals	Patients undergoing PEG (amyotrophic lateral sclerosis: ALS)	In‐depth interviews and group interviews	Themes/sub‐themes: *Patient factors* Acceptance of diagnosis Information sources Patient–carer relationship *Health system factors* Timing of diagnosis Access to resources Interprofessional communication	The results underscored significant obstacles to optimal decision‐making, such as patients' challenges in accepting the diagnosis, dependence on non‐credible information sources and barriers in the patient–carer relationship. Systemic issues, including poor interprofessional communication, limited access to ALS‐specific resources and delays in diagnosis, further complicate the decision‐making process. Clinicians regard teamwork and evidence‐based information as essential facilitators of patient‐centred care. The research determined that it is important to address the gap between the health system's restrictions and the needs of patients to enhance multidisciplinary ALS care.
[26], Malaysia	Qualitative study	17 Healthcare professionals (HCPs)	Patients undergoing PEG	Semi‐structured, in‐depth interviews	Themes: Knowledge of HCPs Communication Understanding among patients Financial and affordability	The results indicated significant barriers to accepting PEG feeding, such as a lack of knowledge among healthcare professionals, inadequate communication within the healthcare team and inadequate understanding among patients and their families regarding the advantages and care associated with PEG feeding. Furthermore, financial constraints were a significant determinant, as numerous patients and their families found PEG insertion prohibitive.
Madigan et al. [27], the United Kingdom	Qualitative study	23 General practitioners (GPs) and nurses	Adult patients undergoing PEG	Semi‐structured interviews	Themes: Previous experiences of enteral feeding Attitude to enteral feeding Problem areas Patient selection Funding issues Discharge information Training needs with respect to enteral feeding	The perception of having inadequate training and frequently receiving no support from secondary care has increased GPs' professional isolation. GPs were often excluded from the initial decision‐making processes regarding patient selection for HETF, which was a significant source of frustration due to the poor communication between primary and secondary care. This exclusion resulted in the management of complicated cases without the necessary knowledge or resources, exacerbating the challenge of providing adequate care.
Martin et al. [28], the United Kingdom	Qualitative study	19 Healthcare professionals (HCPs)	Patients undergoing PEG (amyotrophic lateral sclerosis: ALS)	In‐depth interviews	Themes/sub‐themes: *Patient‐centric factors* Acceptance Perceived effect on quality of life Prolonging life Perception of physical need Practicalities Intervention as signifier Previous experience, knowledge and beliefs Characteristics perceived as helpful or unhelpful in decision‐making *Caregiver and family factors* Caregiver and family motivations Role or involvement Perceived impact on significant others Lack of concordance *HCPs' beliefs, perspectives and actions* Intervention discussions Timing HCP influenced the decision	According to the research, in patients with ALS, perceptions of their condition and concerns about their quality of life had a significant impact on their decision‐making process. Healthcare professionals underscored the significance of preserving patient autonomy, ensuring that the patient's values and preferences informed decision‐making. The research also showed that family members' and caregivers' needs and viewpoints, frequently involved in decision‐making, impacted the decisions. The results indicate that, although patient autonomy is crucial, it is equally essential for healthcare providers to consider the emotional and practical implications of these decisions and the broader family context.
Oliver et al. [29], the United Kingdom	Mixed‐methods (hospice audit and telephone audit)	62 Patient records from 6 hospices and 22 palliative care consultants	Patients undergoing PEG (motor neuron disease: MND)	Hospice audit and structured telephone interviews with consultants	Not applicable (quantitative study)	The research emphasised the significant differences in the utilisation of PEG and NIV for MND patients in hospices throughout the United Kingdom. This variation was associated with the disparity in knowledge and attitudes among palliative care consultants.
Paynter et al. [30], Australia	Mixed‐methods	19 Patients and 15 carers	Patients undergoing PEG (motor neuron diseases)	Semi‐structured interviews ALS functional rating scale (ALSFRS‐R)	Themes: Dimensions of decision‐making Window of opportunity for choice Intrinsic influences on decision‐making Extrinsic influences impacting decision‐making Planning in uncertainty Communication is core	The decision to place a gastrostomy caused significant emotional difficulties for the relatives of patients with neurological disorders. The analysis emphasised the significance of guilt and the impact of medical advice on these decisions. The authors observed that implementing improved support systems and enhanced transparency in communication by healthcare providers could facilitate the elimination of emotional burdens faced by relatives.
Stavroulakis et al. [31], the United Kingdom	Qualitative study (thematic analysis)	10 Patients and 8 carers	Patients undergoing PEG (motor neuron diseases: MND)	Semi‐structured interviews	Themes/sub‐themes: *Factors triggering gastrostomy decision* Prolonged, tiring and effortful meals The task of food preparation Choking and aspiration Weight loss *Factors delaying gastrostomy decision* Reluctance to give up oral feeding Uncertainty over the disease trajectory Not realising the potential benefits Negative perceptions of gastrostomy *Reflections on timing* *Information to support decision‐making*	The progression of the illness, professional advice and clinical recommendations all had an impact on when MND patients had their gastrostomies inserted. The study observed that, despite the potential advantages of early intervention, the decision was frequently postponed due to the disease's complexity and emotional distress. The study indicated that healthcare providers could improve decision‐making by implementing more proactive and transparent communication.
[32], Canada	Quantitative study (Cross‐sectional, exploratory)	17 Nurses	Adult patients undergoing PEG	In‐depth semi‐structured interviews Self‐administered questionnaire	Roles and communication in decision‐making The PEG device Psychosocial/emotional aspects Ethical issues	The study revealed that nurses, despite their close relationships with patients and their families, needed to be more involved in the decision‐making process for PEG. Many nurses felt their input was not sufficiently valued, which led to frustration and emotional stress, particularly when they disagreed with decisions that seemed to prolong life without improving its quality. The study emphasised the importance of including nurses more actively in the decision‐making process, fostering better communication between healthcare providers and ensuring that all healthcare team members are adequately informed about the implications of PEG placement.
[33], The United Kingdom	Qualitative study (hermeneutical and phenomenological)	10 Doctors (5 junior doctors and 5 senior doctors; 5 gastroenterologists and 5 stroke physicians)	Patients undergoing PEG (stroke)	Unstructured, in‐depth interviews	Themes: Task versus process Collaborative working Process of interaction preparation for consent	The decision‐making process was recognised as a complex, multistage procedure requiring meticulous consideration of patient needs and physical assessments rather than a simple task to be completed. The focus has been placed on the importance of collaborative working, particularly in a multidisciplinary team, to ensure that every aspect of the patient's care is considered. The investigation also illustrated that numerous junior physicians perceived themselves as inadequately prepared to provide consent, frequently due to inadequate training in nutritional management and PEG procedures.
Vesey et al. [34], the United Kingdom	Qualitative study	7 Patients	Adult patients undergoing PEG	Semi‐structured interviews	Themes: Information Values Outside pressure Support Reflection	The study's results revealed that patients and their families frequently experienced difficulties in coping with the psychological impacts of making decisions about PEG feeding. Healthcare providers were advised to provide more thorough and compassionate advice to patients and their families during decision‐making.
Van Eenennaam et al. [35], the Netherlands	Qualitative study (thematic analysis)	13 Patients and 12 caregivers	Patients undergoing PEG (amyotrophic lateral sclerosis: ALS)	In‐depth interviews	Theme/sub‐themes: *Feeling of control* Weighing up benefits and concerns. Control in the absence of choice. *Intrinsic factors* Physical necessity Loss and identity Expectations about gastrostomy placement *Extrinsic factors* Family affair Supporting the journey (HCPs)	The psychological challenges caused by the illness significantly impacted the decision‐making process for gastrostomy in patients with ALS. The recurring theme of the conflict between patient autonomy and family involvement was underscored by the significant role played by healthcare provider support in reducing the stress associated with decision‐making.
Yeh et al. [36], Taiwan	Qualitative study (phenomenological approach)	26 Caregivers	Adult patients undergoing PEG	Semi‐structured, in‐depth interviews	Themes: Awareness of suffering Awareness of options Uncertainty Opportunity Contentment with the decision	The caregivers' desire to lessen the patient's suffering had an impact on their decision regarding PEG. The research identified critical themes, including the significance of caregiver satisfaction post‐decision, uncertainty and awareness of options. The caregivers' interactions with healthcare professionals had a big impact on their decisions.

### 2.5 Assessment of Quality

The Qualitative Assessment and Review Tool (QARI) of the Joanna Briggs Institute (JBI), which consists of 10 criteria, was used to assess the quality of these studies and to evaluate the risk of bias. The original research had to meet the following five criteria for inclusion in this systematic review: congruity between research methodology and research objectives, adequate representation of participant quotes in the findings, transparency in data collection and analysis methods, ethical considerations including informed consent and approval when applicable, and relevance and contribution of findings to PEG decision‐making. Quality was measured by assigning a score of 0–2 for each criterion, and studies with a high risk of bias were excluded from the final synthesis.

### 2.6 Ethical Considerations

As this study is a systematic review of published literature, Institutional Review Board (IRB) approval was not required. No human subjects were directly involved, and all data were obtained from publicly available peer‐reviewed studies.

## 3 Results

### 3.1 Search Outcome

**Figure 1:**
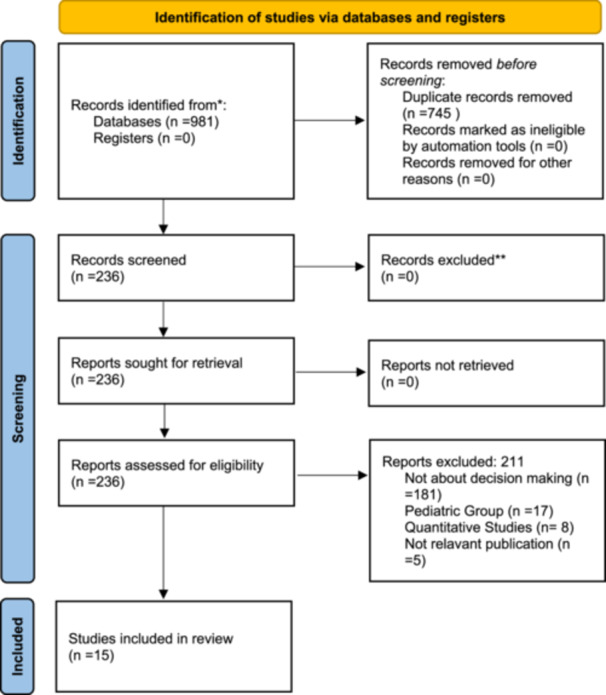
PRISMA flow diagram.

A literature search was performed to identify studies contributing to understanding the decision‐making process for PEG. The initial search yielded a total of 981 articles. After removing 745 duplicate records, 236 articles remained and were subjected to abstract evaluation. From these, 203 were excluded according to the following criteria: 181 studies were solely on intervention or prognosis without reviewing the decision‐making process; 17 studies focused on paediatric populations and did not meet the inclusion criteria; 8 studies were quantitative; and 5 articles were further excluded because they did not focus on the topic. After further assessment, 15 articles qualified—through qualitative analyses—to be part of the final review. Detailed search strategies tailored to each database, including the number of articles identified and search dates, are provided in Table S1. These studies have examined the decision‐making process and the views of health professionals and family members or carers to better understand the factors influencing decisions about PEG (Figure 1 and Table S2).

### 3.2 Study Characteristics

This systematic review and meta‐synthesis included 15 studies examining the decision‐making processes for PEG. The studies were conducted in the following countries: 9 in the United Kingdom [7, 25, 27, 28, 29, 31, 33, 34], 1 in Taiwan [36], 2 in Australia [11, 30] and 3 in other countries (Malaysia, Canada and the Netherlands) [26, 32, 35] (Table 1). Participants in these studies typically included patients requiring enteral nutrition due to conditions such as advanced dementia, ALS, neuromuscular diseases, stroke‐related dysphagia, and head and neck cancers.

The study designs varied, with 12 studies employing qualitative methods, such as semi‐structured interviews, and 3 studies using mixed‐methods approaches. Sample sizes ranged from 7 to 607 participants, reflecting the diversity in research methodologies and population sizes. Table 1 provides detailed information about the studies included in this review. Among the included studies, 53.3% (*n* = 8) focused on ALS or other motor neuron diseases, 6.7% (*n* = 1) examined patients with dementia, 6.7% (*n* = 1) focused on Huntington's disease and 6.7% (*n* = 1) included multiple sclerosis. In terms of participant distribution, 40.0% (*n* = 6) of the studies involved only healthcare professionals, 6.7% (*n* = 1) specifically included only nurses, 20.0% (*n* = 3) focused solely on patients and the remaining 33.3% (*n* = 5) involved mixed groups, including caregivers, family members and patients.

### 3.3 Data Synthesis

Several significant themes regarding factors influencing PEG decision‐making were identified and are presented in Table 2. Emotional burden and psychological distress were commonly reported across studies, particularly among patients, caregivers and families, leading to anxiety, uncertainty, guilt and regret. Ethical considerations and cultural influences emerged as key themes, reflecting tensions between respect for life, quality of life, religious and moral beliefs, and collective family decision‐making.

**Table 2:** Themes and sub‐themes for patients/families/caregivers and healthcare professionals.

Category	Theme	Sub‐Theme
Patient/family/caregiver	1. Emotional and psychological impact of decision‐making	1.1**.** Anxiety and uncertainty in both patients and caregivers 1.2. Guilt and regret in both patients and caregivers
	2. Ethical and moral considerations for both patients and caregivers	2.1. Tension between quality of life and prolonging life, both for patients and caregivers 2.2. Family obligations and collective decision‐making, both for patients and caregivers 2.3. Religious and moral obligations of both patients and caregivers
	3. Communication challenges and information gaps	3.1. Decisional regret in both patients and caregivers 3.2. Conflicting messages from healthcare providers, patients and caregivers
	4. Impact of healthcare professionals on decision‐making	4.1. Supportive and empathetic care for both patients and caregivers 4.2. Perceived abandonment by healthcare providers, both patients and caregivers
Healthcare professionals	1. Ethical and emotional challenges in decision‐making	1.1. Guilt and responsibility 1.2. Emotional burden on health professionals
	2. Communication barriers and conflicting advice	2.1. Difficulty explaining the PEG benefits and risks 2.2. Conflicting opinions among professionals
	3. Professional responsibility and advocacy	3.1. Tension between clinical duty and patient wishes 3.2. Advocacy for patients and families

Communication challenges and information gaps significantly impacted the decision‐making process, as many families reported receiving conflicting information or insufficient guidance from healthcare providers. The role of healthcare professionals was pivotal in shaping PEG decisions. The way healthcare providers communicated prognosis, nutritional needs and PEG alternatives influenced patient and family choices. However, studies also highlighted inconsistencies in physician recommendations, varying levels of patient autonomy and differences in institutional protocols, which contributed to decisional conflict.

Additionally, healthcare professionals' perspectives on PEG varied. Some emphasised patient autonomy and shared decision‐making, while others adopted a more paternalistic approach, particularly when the prognosis was poor. Cultural and contextual differences were also evident, particularly regarding how autonomy was balanced with family involvement and the influence of religious and ethical beliefs on healthcare decisions. The complete categorisation of themes and sub‐themes is provided in Table 2.

## 4 Category 1. Patient/Family/Caregiver

### 4.1 Theme 1: Emotional and Psychological Impact of Decision‐Making

This theme examines the psychological burden that PEG decision‐making imposes on caregivers and patients, particularly in terms of anxiety, guilt and regret. It also explores how these emotional challenges impact both the decision‐makers and their loved ones.

#### 4.1.1 Sub‐Theme 1.1: Anxiety and Uncertainty in Both Patients and Caregivers

Caregivers and patients frequently expressed anxiety, particularly the fear of making the wrong choice regarding PEG. This sub‐theme was identified in six studies, where caregivers and patients commonly expressed concerns about whether they were making the right decision. One patient remarked, ‘*I felt like I was making a decision between life and death, and I wasn't sure if I was doing the right thing’* [6]. A caregiver shared, ‘*Every time I thought about the PEG tube, my heart would race. What if it made things worse?*’ [30].

#### 4.1.2 Sub‐Theme 1.2: Guilt and Regret in Both Patients and Caregivers

Many caregivers and patients described experiencing guilt and regret, especially when the patient's condition worsened after PEG placement. This sub‐theme was explored in five studies, where caregivers and patients often reflected on whether they had made the best possible choice. For example, a caregiver in a study shared, ‘*I keep thinking, what if we made the wrong choice? What if there was something else, we could have done?’* [30]. A patient expressed, ‘*I regret agreeing to the PEG. I feel like I might have caused more suffering, even though I thought I was helping’* [34]. These emotions underscore the long‐term psychological impact of PEG‐related decisions.

### 4.2 Theme 2: Ethical and Moral Considerations for Both Patients and Caregivers

This theme explored how cultural values, ethical dilemmas and moral perspectives influenced PEG decision‐making. Across multiple studies, caregivers and patients described struggling with the balance between sustaining life and ensuring quality of life, as well as the role of family and religious beliefs in shaping their choices.

#### 4.2.1 Sub‐Theme 2.1: Tension Between Quality of Life and Prolonging Life, Both for Patients and Caregivers

In several studies, caregivers and patients faced the ethical dilemma of prolonging life versus prioritising the patient's quality of life. This sub‐theme appeared in seven studies, where caregivers and patients expressed concerns about prolonging suffering for the sake of life preservation, while others emphasised the sacredness of life. A caregiver in a study stated, ‘*We had to do everything we could to keep my father alive, even if it meant more suffering’* [36]. One patient reflected, ‘*I wasn't sure how much longer I could endure, but everyone around me believed choosing the PEG meant choosing hope’* [30].

#### 4.2.2 Sub‐Theme 2.2: Family Obligations and Collective Decision‐Making, Both for Patients and Caregivers

In five studies, decision‐making was reported as a collective process influenced by family expectations and social obligations. Caregivers and patients described feeling pressured by cultural norms to continue medical interventions, even when personal beliefs conflicted with family expectations. For instance, a caregiver in the study mentioned, ‘*There's an unspoken rule here that you don't give up on life. It's not easy to talk about letting go when everyone expects you to keep trying’* [31]. One patient said, ‘*In our family, decisions like this aren't just about the patient; they're about what the whole family believes is right’* [35]. This sub‐theme illustrates the cultural pressures that can complicate ethical decision‐making.

#### 4.2.3 Sub‐Theme 2.3. Religious and Moral Obligations of Both Patients and Caregivers

In four studies, caregivers and patients described how religious beliefs shaped their approach to PEG decision‐making. Religious values were reported as an important guiding principle in some cases, particularly when viewing life‐sustaining interventions as a moral duty. A caregiver in a study shared, ‘*In our culture, we believe it's our duty to keep our loved ones alive as long as possible. Not doing everything we could feel like a betrayal*’ [31]. A patient expressed, ‘*I believed that accepting the PEG was part of honoring the life given to us—it wasn't an easy decision, but my beliefs guided me*’ [35]. This sub‐theme illustrates how religious and moral obligations can influence decision‐making.

### 4.3 Theme 3. Communication Challenges and Information Gaps

This theme illustrated caregivers' and patients' difficulties in obtaining clear, direct information from healthcare professionals. It highlighted the communication breakdown that can lead to confusion and uncertainty during decision‐making.

#### 4.3.1 Sub‐Theme 3.1. Decisional Regret in Both Patients and Caregivers

Several studies highlighted that caregivers and patients often felt they did not receive enough information to make an informed decision. This sub‐theme was reported in eight studies, where caregivers and patients expressed frustration over the lack of clear and comprehensive information. A caregiver in the study noted, ‘*They told me it was a routine procedure, but no one mentioned the complications’* [6]. A patient mentioned, ‘*I wasn't told about the long‐term care involved. If I had known, I might have made a different choice’* [30]. This sub‐theme underscores the frustration and confusion that arise when essential information is not provided.

#### 4.3.2 Sub‐Theme 3.2. Conflicting Messages From Healthcare Providers, Patients and Caregivers

Caregivers and patients frequently reported receiving conflicting information from different healthcare providers, which exacerbated their uncertainty. This sub‐theme was discussed in six studies, where caregivers and patients described how conflicting advice made it difficult to make informed decisions. A caregiver shared, ‘*We got different opinions from every doctor we spoke to. It was overwhelming and left us more confused than when we started’* [7]. A patient echoed this sentiment, saying, ‘*One doctor said it was necessary, another said it wasn't. We didn't know who to believe’* [25]. This sub‐theme highlights the need for consistent and coordinated communication among healthcare teams.

### 4.4 Theme 4. Impact of Healthcare Professionals on Decision‐Making

The theme has identified that health professionals are considered critical in the decision to PEG. It talks about supportive roles and challenges in their care.

#### 4.4.1 Sub‐Theme 4.1. Supportive and Empathetic Care for Both Patients and Caregivers

Positive experiences were reported when healthcare professionals took the time to explain options thoroughly and provided empathetic support. This sub‐theme was emphasised in seven studies, where caregivers and patients described how empathetic care from healthcare providers helped them navigate the decision‐making process. A patient in Eenennaam et al.'s (2023) study expressed, ‘*They didn't rush us. They took the time to explain everything and made sure we understood what we were agreeing to*’ [35]. A caregiver noted, ‘*The doctors and nurses were so patient with us. They really listened to our concerns and helped us make the best decision for my mom*’ [7]. This sub‐theme emphasises the importance of patient‐centred care in the decision‐making process.

#### 4.4.2 Sub‐Theme 4.2. Perceived Abandonment by Healthcare Providers, Both Patients and Caregivers

Conversely, some caregivers and patients felt abandoned when healthcare providers did not respect their decisions or provide adequate support. This sub‐theme was reported in four studies, where caregivers and patients described feelings of neglect after making decisions that healthcare providers did not fully support. In Paynter et al.'s study, a caregiver lamented, ‘*We felt abandoned by the doctors and nurses. Once we decided not to go ahead with the PEG, it was like they washed their hands of us’* [30]. A patient shared, ‘*When we refused the PEG, the tone changed. It was like we were no longer important to them’* [29]. This sub‐theme reflects the negative impact of insufficient support from healthcare professionals.

## 5 Category 2. Healthcare Professionals

While the experiences of patients and caregivers in the decision‐making process have been discussed, the role and challenges faced by healthcare professionals are equally important. Below, the themes related to healthcare professionals' experiences are explored.

### 5.1 Theme 1: Ethical and Emotional Challenges in Decision‐Making

This theme discusses the significance of ethical dilemmas and emotional stresses that healthcare professionals face when guiding patients and families through the PEG decision‐making process. These discussions often involve balancing life prolongation and quality of life, leading to emotional distress and ethical conflict.

#### 5.1.1 Sub‐Theme 1.1: Guilt and Responsibility

Among health professionals, but especially in decisions about PEG, life prolongation was often weighed against protecting quality of life (four studies). Martin et al. [28] described how professionals struggled with whether their recommendations truly benefited patients. A healthcare provider reflected, ‘*I often wonder whether we are prolonging life or suffering. It is difficult to tell the family that the procedure might not improve their loved one's quality of life.’*

Additionally, they felt responsible for families' expectations, especially when they had high hopes for PEG. Furthermore, the degree of responsibility became deeper when families held high hopes that the intervention would be successful. Todd et al. [32] also commented, ‘*The emotional toll of seeing families hold onto hope, even though we know the reality may not turn out to be the way they are hoping it will, is tough.’*

#### 5.1.2 Sub‐Theme 1.2: Emotional Burden on Health Professionals

Healthcare professionals who collaborated closely with families and patients in PEG decision‐making experienced significant emotional strain. If the patients had been under care for a long time or were suffering from severe, usually terminal diseases, then the emotional consequence of such decisions was very great (five studies). Hogden et al. [11] showed emotional exhaustion in nurses due to these decisions. ‘*It is draining emotionally to see the families having to consider such a decision, mainly when we already know that the outcome may not be as they wish,’* said one nurse.

These decisions affected the patient, the family and the emotional resilience of the healthcare professionals as they added to their role challenges.

### 5.2 Theme 2: Communication Barriers and Conflicting Advice

Effective communication is crucial in decision‐making, yet healthcare professionals often face significant barriers in conveying clear and consistent information about PEG. This theme examines the difficulties in providing comprehensive information and the impact of conflicting advice among medical teams.

#### 5.2.1 Sub‐Theme 2.1: Difficulty in Explaining the PEG Benefits and Risks

Health professionals reported challenges in effectively communicating all the benefits and risks of the PEG to patients (five studies). As such, Madigan et al. [27] showed that professionals often felt they needed to spend more time and resources fully explaining to the patients and their families, resulting in misunderstandings and further complications in the decision‐making process. As one of the healthcare workers noted, ‘*It is tough to tell the families that yes, this could prolong the child's life, but it is not necessarily going to enhance the quality, and sometimes that is a hard concept for them to grasp.’*

These communication difficulties were especially pronounced when decisions involved the patient's quality of life, making it a critical issue that healthcare professionals frequently faced.

#### 5.2.2 Sub‐Theme 2.2: Conflicting Opinions Among Professionals

Another significant problem was the inconsistent advice different healthcare professionals gave, which further complicated family decisions (five studies). Trautner [33] said that this not only hurt the people who needed help; inconsistency in the medical opinions had left the families questioning what they were doing and what kind of trust would be possible. Affected families exclaimed, ‘*The conflicting opinions among the medical team left families feeling uncertain about the best course of action.’*

These varying clinical perspectives and mixed messages created additional layers of confusion for patients and their families, ultimately making PEG decision‐making more challenging.

### 5.3 Theme 3: Professional Responsibility and Advocacy

In addition to clinical practice, many healthcare professionals also played a key role in advocating for the needs of patients and their families. This theme captures the dual responsibility of healthcare workers: providing medical guidance while respecting patient preferences. It also reflects the emotional support they must give families during difficult healthcare decisions, positioning them as crucial intermediaries between clinical duty and compassionate care.

#### 5.3.1 Sub‐Theme 3.1: Tension Between Clinical Duty and Patient Wishes

It has always been a challenge for healthcare professionals to balance clinical duties with respect for patient autonomy (four studies). According to Jaafar et al. [26], healthcare providers aimed to offer medical recommendations while ensuring that patient autonomy was prioritised, particularly when patients were unable to make informed decisions. One healthcare provider said, ‘*As healthcare providers, we are often caught in that delicate balance of trying to weigh clinical obligations with patient autonomy in a manner that upholds the dignity of the patient's wishes while ensuring their safety.’*

This fragile balance became even more complex when patients were unable to express their preferences clearly, further complicating the decision‐making process.

#### 5.3.2 Sub‐Theme 3.2: Advocacy for Patients and Families

Healthcare professionals also recognised their role as essential advocates for patients and families (five studies). According to Martin et al. [28], during decision‐making, professionals not only provided medical advice but also played a crucial role in addressing the emotional and informational needs of families. One health professional shared, *‘Families look to us for medical advice but emotional support in guiding these tough decisions.’*

This advocacy role emphasised the broader responsibilities of healthcare professionals, ensuring that both medical and emotional needs were met. Such efforts contributed to a more holistic and patient‐centred approach to PEG decision‐making.

### 5.4 Assessment of Quality

Table 3 shows the quality assessment of the included studies. In the evaluation of the qualitative studies, it was seen that most of the studies were of high quality and thus received full marks within most criteria. Mainly, it was observed that the data collection tools and analysis procedures were described systematically, with the findings supporting the results. Some studies needed more specific criteria, such as those by Stavroulalis et al. (2014) and Hogden et al. [11], that required establishing a connection to the theoretical framework. In contrast, others, such as Jaafar et al. [26] and Clarke et al. [7], did not indicate the sampling strategy. Despite these deficiencies, most studies had rather precise and reliable processes for collecting and analysing data, which means the results generally had robustness.

**Table 3:** Quality appraisal using the JBI critical appraisal checklist for qualitative research.

Reporting criteria	Yeh et al. [36]	Trautner [33]	Todd et al. [32]	Oliver et al. [29]	Stavroulakis et al. [31]	Madigan et al. [27]	Brotherton et al. [6]	van Eenennaam et al. [35]	Hogden et al. [11]	Greenaway et al. [25]	Vesey et al. [34]	Martin et al. [28]	Paynter et al. [30]	Jaafar et al. [26]	Clarke et al. [7]
Question/objective sufficiently described?	2	2	2	2	2	2	2	2	2	2	2	2	2	2	2
Study design evident and appropriate?	2	2	2	2	2	2	2	2	2	2	2	2	2	2	2
Context for the study clear?	1	2	2	2	2	2	2	2	2	2	2	1	2	2	2
Connection to a theoretical framework/wider body of knowledge?	1	1	2	2	1	2	2	1	2	2	2	1	2	2	1
Sampling strategy described, relevant and justified?	1	1	2	2	1	2	2	1	2	2	2	1	2	2	1
Data collection methods clearly described and systematic?	2	2	2	2	2	2	2	2	2	2	2	2	2	2	2
Data analysis clearly described and systematic?	2	2	2	2	2	2	2	2	2	2	2	2	2	2	2
Use of verification procedure(s) to establish credibility?	2	1	2	2	2	2	2	1	2	2	2	2	2	2	2
Conclusions supported by the results?	2	2	2	2	2	2	2	2	2	2	2	2	2	2	2
Reflexivity of the account?	1	2	2	2	1	2	2	2	2	2	2	1	2	2	1

Nevertheless, more linking to the theoretical framework is needed, and improving sampling strategies was suggested for future studies. However, the clarity of the data collection and analysis procedures outweighs these shortcomings, so these data are generally of good quality.

## 6 Discussion

The decision‐making process for PEG involves medical, emotional, ethical and cultural factors. The results from this study provide insight into the multiple challenges that patients, caregivers and healthcare professionals face when making these critical decisions. Additionally, this study examined how these themes align with, expand upon or challenge existing literature on PEG and its implications for clinical practice.

This study highlighted the emotional burden of decision‐making regarding PEG as a critical issue for patients, caregivers and healthcare professionals. Feelings of anxiety, uncertainty, guilt and regret reported by participants align with existing research on decision‐making in critical care settings. Winterbottom et al. [37] noted that decision‐making in uncertain situations often results in significant emotional distress, particularly when outcomes are unpredictable. Henao et al. [38] further emphasised racial disparities in PEG prevalence among patients with advanced dementia, demonstrating how these disparities add complexity to the decision‐making process. The perception of PEG as an irreversible decision, which caregivers frequently interpret as a choice between life and death, further exacerbates distress [39, 40]. This suggests the need for structured emotional support interventions, such as dedicated counselling services, peer‐support programmes and healthcare provider training focused on delivering compassionate communication [41, 42].

Another significant issue explored in this study was the ethical challenges associated with PEG, particularly the conflict between prolonging life and ensuring quality of life. This dilemma is widely discussed in bioethics, often framed as a tension between beneficence (promoting well‐being) and non‐maleficence (avoiding harm) [43]. The studies included in this systematic review demonstrated how these ethical principles were applied in the context of PEG, often conflicting with cultural and religious beliefs that shape perceptions of what constitutes a ‘good’ decision regarding life‐sustaining treatments. Fessler et al. [44] also found that disagreements among healthcare professionals regarding the appropriateness of PEG frequently intensified ethical dilemmas. Such conflicts can lead to additional stress for patients, caregivers and healthcare professionals seeking guidance during these complex decisions [45, 46]. To mitigate these ethical conflicts, the development of shared decision‐making frameworks that incorporate ethical guidelines, advanced care planning and multidisciplinary case discussions may be beneficial.

Many studies in this systematic review, particularly those conducted in collectivist cultures, highlighted how family obligations and religious beliefs strongly influenced PEG decision‐making. In cultures where family unity and collective well‐being are prioritised, decisions about life‐sustaining treatments often reflect these broader cultural values [47]. Komiya et al. [48] found that in Japan, a collectivist society, PEG usage has been declining, reflecting a shift in both cultural and medical perspectives on quality of life and end‐of‐life care. These findings align with those of Constantinou et al. [49], who argue that cultural values play a fundamental role in shaping how patients and families approach healthcare decisions. These insights emphasise the importance of culturally sensitive approaches in clinical practice, ensuring that healthcare providers respect individual autonomy while also acknowledging the cultural and ethical contexts influencing PEG decision‐making. In this context, healthcare professionals should develop cultural competence, which includes engaging in open and respectful discussions with patients and families, gaining insight into their cultural values and beliefs, and collaborating with culturally sensitive mediators or interpreters when necessary [47, 49, 50]. Future studies should explore culturally adapted decision‐making models that consider religious, familial and social perspectives while promoting patient autonomy.

The communication challenges and information gaps identified in this study align with existing literature that underscores the importance of clear, consistent and transparent communication in healthcare. Studies have reported that communication breakdowns are a significant barrier to shared decision‐making, often leading to confusion and decisional conflict [51, 52]. A key finding was that healthcare professionals sometimes provided conflicting advice regarding PEG, further complicating decision‐making for patients and caregivers. Effective communication in healthcare settings goes beyond merely providing information; it also involves ensuring that information is clearly understood and that patients and caregivers feel adequately supported throughout the decision‐making process. To address these challenges, standardised communication protocols and decision aids (such as visual tools and educational pamphlets) could be implemented to ensure that patients and caregivers receive comprehensive and consistent information about PEG. To improve communication, healthcare professionals should receive targeted training in communication techniques that enhance clarity, consistency and empathy [53].

Another essential theme in this study was the role of healthcare professionals as advocates for patients and families during the PEG decision‐making process. A well‐structured discharge planning process should begin during hospital admission, ensuring that patients and their caregivers receive continuous education on PEG management, complication prevention and emergency response strategies throughout hospitalisation. Establishing a strong provider–caregiver relationship is crucial to increasing patient engagement and reducing distress associated with PEG care at home.

Recent literature emphasises that structured educational programmes positively influence patient and family decision‐making regarding PEG. For instance, Santos et al. [54] found that in‐hospital education and telemonitoring interventions significantly improved caregivers' competence in managing gastrostomy [54]. Similarly, Despain et al. [55] found that PEG patients often lack adequate follow‐up post‐discharge, highlighting the need for structured educational interventions [55]. Sezer et al. [56] demonstrated that caregivers frequently struggle with PEG management at home, reinforcing the necessity of comprehensive training and follow‐up care [56]. Additionally, mobile health interventions have been shown to improve self‐care ability and reduce complications among PEG patients, as evidenced by Chen et al. [57]. Such training programmes can enhance confidence in decision‐making and alleviate anxiety.

Therefore, educational interventions should adopt a multistage approach, including in‐person hospital training, hands‐on skill sessions and post‐discharge telemonitoring to ensure continuous support. Studies indicate that many patients and caregivers feel unprepared for post‐discharge PEG management, leading to anxiety and avoidable complications [58]. To address this gap, pre‐discharge hands‐on training sessions should be implemented, focusing on PEG handling, recognising early signs of complications and knowing when to seek medical assistance. Additionally, post‐discharge follow‐up services such as telehealth support, home visits and scheduled check‐ins could further support caregivers and improve patient outcomes by reducing preventable complications and reinforcing proper PEG care practices [9]. Many of the reviewed studies underscored the importance of compassionate and respectful care, with patients and caregivers reporting better outcomes when healthcare professionals provided clear explanations and emotional support. This aligns with the patient‐centred care model [59], which emphasises the necessity of meaningful interactions between healthcare providers, patients and caregivers. However, this study also highlighted instances where caregivers felt abandoned by their healthcare providers after making the decision for PEG, particularly when they did not receive adequate follow‐up support. According to existing literature, ongoing support and maintaining a positive relationship with patients and caregivers are crucial, even in cases where healthcare professionals may not fully agree with the final decision regarding PEG [60, 61]. Developing interdisciplinary PEG consultation teams and follow‐up services for patients and families could ensure continued support, reducing feelings of abandonment and decisional regret.

### 6.1 Limitations and Future Research

While this study provides valuable insights into the decision‐making processes surrounding PEG, certain limitations must be acknowledged. One major limitation is that only English and Turkish‐language studies were included, which may have introduced linguistic and cultural bias by excluding perspectives from non‐English‐speaking populations. Additionally, although this review incorporates studies from different cultural settings, its findings may not be universally generalisable, as cultural attitudes towards PEG and end‐of‐life care can vary significantly. Another limitation is that the included studies focused primarily on qualitative data, meaning that findings are context‐specific and may not be directly applicable to all patient populations.

Despite these limitations, this study has several strengths. It is one of the few systematic reviews to synthesise qualitative evidence on the emotional, ethical and cultural dimensions of PEG decision‐making. The inclusion of multiple perspectives—patients, caregivers and healthcare professionals—enhances the depth and richness of the findings, providing a comprehensive understanding of the challenges involved in PEG‐related decisions.

Future research should explore decision‐making in more diverse healthcare systems and cultural contexts to improve the generalisability of findings. Further studies are also needed to assess the long‐term psychological impact of PEG decisions on patients and caregivers, as well as to evaluate the effectiveness of various communication strategies in reducing decisional conflict.

### 6.2 Implications for Nursing

The findings from this systematic review and meta‐synthesis emphasise the critical role of nurses in supporting patients, families and caregivers during the decision‐making process for PEG. Nurses should prioritise clear and consistent communication to reduce the confusion and decisional conflict often experienced by patients and caregivers. Training in communication skills that focus on empathy, cultural sensitivity and shared decision‐making is essential for fostering patient‐centred care. Moreover, nurses should be well‐prepared to recognise and address the emotional burden that arises during PEG decision‐making, including feelings of anxiety, guilt and regret. Providing emotional support through counselling services, support groups and compassionate communication can significantly alleviate the psychological distress of patients and caregivers. Cultural competence is another key implication for nursing practice. Nurses must be tuned to the cultural and religious values that shape patients' and families' decisions regarding PEG and other life‐sustaining treatments. Integrating cultural mediators or interpreters into care teams can facilitate better understanding and respect for patients' cultural backgrounds, leading to more informed and culturally appropriate decision‐making.

Furthermore, the advocacy role of nurses is highlighted in ensuring patients and families feel supported throughout the entire decision‐making journey—not only during the initial discussions but also in the follow‐up stages. Nurses should maintain positive, ongoing relationships with caregivers, even when differing perspectives arise regarding PEG. This includes offering ongoing support after the decision has been made, as caregivers often feel abandoned by healthcare professionals once a decision is finalised. Ensuring patients and their families receive continuous, holistic care that incorporates emotional, ethical and cultural considerations will enhance the quality of care and outcomes for PEG patients.

## 7 Conclusion

PEG decision‐making is a complicated and multidimensional process that considers the communicative, emotional, ethical and cultural aspects. The results of this study have significant implications for clinical practice. First, there is an obvious need for a far more coordinated and consistent communication strategy to ensure patients and carers get the information they want in a timely fashion and in an understandable format. This includes transparent, consistent and culturally appropriate information about the advantages of PEG, associated disadvantages and alternatives to its implantation. Training health professionals in shared decision‐making methodologies that highlight empathy, active listening and engaging patients in decisions regarding their care is necessary. Second, cultural sensitivity is critical for dealing with a diverse patient population. Healthcare professionals must be able to guide patients in a way that respects their values and considers the family's cultural context while navigating ethical and cultural decision‐making issues. This could entail assessing the cultural and religious beliefs that influence patients' healthcare decisions and working with cultural mediators or interpreters. The psychological burden that caregivers face should also be considered. Providing additional assistance, such as therapy or support groups, may alleviate some of the emotional pain caused by these choices.

## Author Contributions

**Hande Nur Arslan:** conceptualisation, writing – review and editing, writing – original draft, data curation, methodology, formal analysis, software, visualisation, resources. **Gamze Bozkul:** data curation, validation, writing – original draft, writing – review and editing. **Sevilay Şenol Çelik:** formal analysis, methodology, project administration, resources, software, writing – review and editing.

## Ethics Statement

The author(s) affirm that the methods used in the data analyses are suitably applied to their data within their study design and context, and the statistical findings have been implemented and interpreted correctly.

## Conflicts of Interest

The authors declare no conflicts of interest.

## Supporting information

Search Strategy Table.

Excluded final.

## Data Availability

The authors have nothing to report.
