# Supplementary material for: Emotional, Ethical and Cultural Challenges in Percutaneous Endoscopic Gastrostomy (PEG) Decision‐Making: A Systematic Review and Meta‐Synthesis
Source: Health Expect. 2026 Apr 24;29(2):e70294. doi: 10.1111/hex.70294 (PMC13108423; doi:10.1111/hex.70294)
Supplement: Supplementary file 1 — Search Strategy Table. [file HEX-29-e70294-s002.docx]

# Supplementary Table 1. Search Strategies and Number of Articles Identified from Each Database (September–October 2024)

| Database | Search Strategy | Articles Identified | Search Date |
| --- | --- | --- | --- |
| PubMed | ("Percutaneous Endoscopic Gastrostomy"[MeSH] OR "PEG tube" OR "feeding tube" OR "enteral feeding") AND ("decision-making"[MeSH] OR "shared decision-making" OR "informed consent" OR "patient preference" OR "caregiver decision-making") AND ("qualitative research" OR "thematic synthesis" OR "meta-synthesis" OR "interviews") | 312 | September 25, 2024 |
| CINAHL | ("PEG feeding"[CINAHL Headings] OR "Enteral Nutrition" OR "long-term tube feeding" OR "nutritional support") AND ("patient experience" OR "caregiver burden" OR "decision-making process" OR "family involvement") AND ("qualitative methods" OR "phenomenology" OR "grounded theory" OR "content analysis") | 184 | September 28, 2024 |
| Scopus | ("Percutaneous Endoscopic Gastrostomy" OR "PEG decision-making" OR "feeding tube placement") AND ("qualitative study" OR "patient-centered decision-making" OR "healthcare provider perspective" OR "family and caregiver perspective") AND ("thematic analysis" OR "meta-synthesis" OR "systematic review") | 205 | October 2, 2024 |
| Web of Science | ("Gastrostomy" AND "decision-making") OR ("feeding tube ethics") AND ("patient and family perspective" OR "care team communication" OR "medical ethics") AND ("qualitative synthesis" OR "evidence-based review") | 134 | October 5, 2024 |
| MEDLINE | ("PEG"[MeSH] OR "feeding tube" OR "artificial nutrition") AND ("shared decision-making"[MeSH] OR "ethical concerns" OR "family perspectives") AND ("qualitative synthesis" OR "patient narratives" OR "physician-family communication") | 89 | October 8, 2024 |
| TRDizin | ("PEG beslenme" OR "enteral beslenme" OR "gastrostomi tüpü") AND ("karar süreci" OR "hasta ve bakım veren deneyimi" OR "multidisipliner yaklaşım") AND ("nitel araştırma" OR "tematik analiz") | 57 | October 10, 2024 |
