# Supplementary material for: Emotional, Ethical and Cultural Challenges in Percutaneous Endoscopic Gastrostomy (PEG) Decision‐Making: A Systematic Review and Meta‐Synthesis
Source: Health Expect. 2026 Apr 24;29(2):e70294. doi: 10.1111/hex.70294 (PMC13108423; doi:10.1111/hex.70294)
Supplement: Supplementary file 2 — Excluded final. [file HEX-29-e70294-s001.docx]

**Supplementary Table 2.** Full-text Studies Reviewed and Reasons for Exclusion

| **Title** | **Author** | **Publication Year** | **Publication Title** | **Exclusion Reason** |
| --- | --- | --- | --- | --- |
| **Carers’ experiences of home enteral feeding: A survey exploring medicines administration challenges and strategies** | Alsaeed, D.; Furniss, D.; Blandford, A.; Smith, F.; Orlu, M. | 2018 | Journal of Clinical Pharmacy and Therapeutics | Not about decision making |
| **Patients and home carers' experience and perceptions of different modalities of enteral feeding** | Ang, Shin Yuh; Lim, Mei Ling; Ng, Xin Ping; Lam, Madeleine; Chan, Mei Mei; Lopez, Violeta; Lim, Siew Hoon | 2019 | Journal of Clinical Nursing | Quantitative Studies |
| **Acceptability and outcomes of the Percutaneous Endoscopic Gastrostomy (PEG) tube placement- patients' and care givers' perspectives** | Anis, Muhammad K; Abid, Shahab; Jafri, Wasim; Abbas, Zaigham; Shah, Hasnain A; Hamid, Saeed; Wasaya, Rozina | 2006 | BMC Gastroenterology | Pediatric Group |
| **Home enteral nutrition: Use of photo‐elicitation to capture patient and caregiver experiences** | Asiedu, Gladys B.; Carroll, Katherine; Griffin, Joan M.; Hurt, Ryan T.; Mundi, Manpreet | 2018 | Health Science Reports | Pediatric Group |
| **Feeding Tubes for Older People With Advanced Dementia Living in the Community in Israel** | Bentur, Netta; Sternberg, Shelley; Shuldiner, Jennifer; Dwolatzky, Tzvi | 2015 | American Journal of Alzheimer's Disease & Other Dementias® | Pediatric Group |
| **Struggling in an inescapable life situation: being a close relative of a person dependent on home enteral tube feeding** | Bjuresäter, Kaisa; Larsson, Maria; Athlin, Elsy | 2012 | Journal of Clinical Nursing | Not about decision making |
| **Determination of Problems of the Patients Depending on Enteral Tube Feeding at Home and Their Interventions for Those Problems** | Ci̇ğeroğlu, R Dilek BOZ; Karadağ, Ayişe | 2009 | Hemşirelikte Araştırma  Geliştirme Dergisi | Quantitative Studies |
| **The impact of percutaneous endoscopic gastrostomy feeding upon daily life in adults** | Brotherton, A.; Abbott, J.; Aggett, P. | 2006 | Journal of Human Nutrition and Dietetics | Quantitative Studies |
| **Home percutaneous endoscopic gastrostomy feeding: perceptions of patients, carers, nurses and dietitians** | Brotherton, Ailsa M.; Abbott, Janice; Hurley, Margaret A.; Aggett, Peter J. | 2007 | Journal of Advanced Nursing | Not about decision making |
| **Percutaneous Endoscopic Gastrostomy Feeding in Nursing Homes: Relatives' Perceptions** | Brotherton, Ailsa M.; Carter, Bernie | 2007 | Clinical Nursing Research | Not about decision making |
| **Enteral feeding in motor neurone disease: Patients’ perspectives and impact on quality of life** | Chhetri, Suresh Kumar; Bradley, Belinda Fay; Majeed, Tahir; Lea, Robert William | 2017 | Palliative Medicine | Not about decision making |
| **Dilemmas for guardians of advanced dementia patients regarding tube feeding** | Gil, Efrat; Agmon, Maayan; Hirsch, Ayal; Ziv, Miriam; Zisberg, Anna | 2018 | Age and Ageing | Not relevant publication |
| **The experiences and support needs of people living at home with an enteral tube: a qualitative interview study** | Green, S. M.; Townsend, K.; Jarrett, N.; Fader, M. | 2019 | Journal of Human Nutrition and Dietetics | Quantitative Studies |
| **People with enteral tubes and their carers' views of living with a tube and managing associated problems: A qualitative interview study** | Green, Sue M.; Townsend, Kay; Jarrett, Nikki; Westoby, Catherine; Fader, Mandy | 2019 | Journal of Clinical Nursing | Not relevant publication |
| **Percutaneous endoscopic gastrostomies: the burden of treatment from a patient perspective** | Jordan, Sue; Philpin, Sue; Warring, Joanne; Cheung, Wai Yee; Williams, John | 2006 | Journal of Advanced Nursing | Pediatric Group |
| **Home Enteral Nutrition therapy: Difficulties, satisfactions and support needs of caregivers assisting older patients** | Jukic P, Nikolina; Gagliardi, Cristina; Fagnani, Donata; Venturini, Claudia; Orlandoni, Paolo | 2017 | Clinical Nutrition | Quantitative Studies |
| **Gastrostomies Preserve But Do Not Increase Quality of Life for Patients and Caregivers** | Kurien, Matthew; Andrews, Rebecca E.; Tattersall, Rachel; McAlindon, Mark E.; Wong, Emma F.; Johnston, Alex J.; Hoeroldt, Barbara; Dear, Keith L.; Sanders, David S. | 2017 | Clinical Gastroenterology and Hepatology | Pediatric Group |
| **The Experiences of Patients With Advanced Head and Neck Cancer With a Percutaneous Endoscopic Gastrostomy Tube: A Qualitative Descriptive Study** | Kwong, Janna P. Y.; Stokes, Edith J.; Posluns, Elaine C.; Fitch, Margaret I.; McAndrew, Alison; Vandenbussche, Katherine A. | 2014 | Nutrition in Clinical Practice | Pediatric Group |
| **The impact of home enteral tube feeding in everyday life: a qualitative study: Home enteral tube feeding** | Liley, Anita J.; Manthorpe, Jill | 2003 | Health & Social Care in the Community | Quantitative Studies |
| **Patients’ perspectives of living with a percutaneous endoscopic gastrostomy (PEG)** | Martin, Lena; Blomberg, John; Lagergren, Pernilla | 2012 | BMC Gastroenterology | Not relevant publication |
| **Different experiences and perspectives between head and neck cancer patients and their care‐givers on their daily impact of a gastrostomy tube** | Mayre‐Chilton, K. M.; Talwar, B. P.; Goff, L. M. | 2011 | Journal of Human Nutrition and Dietetics | Not about decision making |
| **Head and neck cancer patients' experiences of percutaneous endoscopic gastrostomy feeding: a Q-methodology study: Cancer patients' experiences of PEGs** | Merrick, S.; Farrell, D. | 2012 | European Journal of Cancer Care | Not relevant publication |
| **The Experience of Head and Neck Cancer Patients With a Percutaneous Endoscopic Gastrostomy Tube at a Canadian Cancer Center** | Osborne, Joanna B.; Collin, Laura A.; Posluns, Elaine C.; Stokes, Edith J.; Vandenbussche, Katherine A. | 2012 | Nutrition in Clinical Practice | Not relevant publication |
| **A Matter of Taste? Quality of Life in Day-to-Day Living with ALS and a Feeding Tube** | Pols, Jeannette; Limburg, Sarah | 2016 | Culture, Medicine, and Psychiatry | Pediatric Group |
| **Home Percutaneous Endoscopic Gastrostomy Feeding: Difficulties and Needs of Caregivers, Qualitative Study** | Sezer, Rana Elcin; Ozdemir Koken, Zeliha; Senol Celik, Sevilay | 2020 | Journal of Parenteral and Enteral Nutrition | Not about decision making |
| **Percutaneous venting gastrostomy/gastrojejunostomy for malignant bowel obstruction: a qualitative study** | Singh Curry, Rebecca; Evans, Elizabeth; Raftery, Anne-Marie; Hiscock, Julia; Poolman, Marlise | 2019 | BMJ Supportive & Palliative Care | Quantitative Studies |
| **The impact of gastrostomy in motor neurone disease: challenges and benefits from a patient and carer perspective** | Stavroulakis, Theocharis; Baird, Wendy O; Baxter, Susan K; Walsh, Theresa; Shaw, Pamela J; McDermott, Christopher J | 2016 | BMJ Supportive & Palliative Care | Not relevant publication |
